# The frequency of rehospitalization and associated factors in Colombian psychiatric patients: a cohort study

**DOI:** 10.1186/1471-244X-14-161

**Published:** 2014-06-02

**Authors:** Luis Eduardo Jaramillo-Gonzalez, Ricardo Sanchez-Pedraza, Maria Isabel Herazo

**Affiliations:** 1 Department of Psychiatry, National University of Colombia, Faculty of Medicine, Office 202, Bogotá, Colombia; 2 Clinical Psychiatrist, Hospital San Rafael, Pasto, Colombia

**Keywords:** Length of stay/statistics & numerical data, Mental disorders/epidemiology, Patient readmission/statistics & numerical data, Regression analysis, Risk factors

## Abstract

**Background:**

The rehospitalization of patients with mental disorders is common, with rehospitalization rates of up to 80% observed in these patients. This phenomenon negatively impacts families, patients, and the health care system. Several factors have been associated with an increased likelihood of rehospitalization. This study was aimed at determining the frequency and the factors associated with rehospitalization in a psychiatric clinic.

**Methods:**

We performed a prospective cohort study with 361 patients who were hospitalized at the Clinic of Our Lady of Peace in Bogota, Colombia from August-December 2009. We calculated the incidence rates of rehospitalization and the risk factors using Cox regression.

**Results:**

Overall, 60% of the patients in this cohort were rehospitalized during the year that followed the index event. The variables associated with rehospitalization were separated, divorced, or single status; higher socio-economic strata; a longer duration of index hospitalization; and a diagnosis of substance abuse, schizophrenia, bipolar disorder, or depression.

**Conclusions:**

The rehospitalization rate in our study was as high as reported in other studies. The associated factors with it in this group, may contribute to the design of programs that will reduce the frequency of rehospitalization among patients with mental disorders, in countries like Colombia. Additionally, these results may be useful in interventions, such as coping skills training, psycho-education, and community care strategies, which have been demonstrated to reduce the frequency of rehospitalization.

## Background

Rehospitalization is frequent among patients with mental disorders, especially because health care systems advocate for short hospital stays [1]. Moreover, several studies have found that readmitted patients [2] account for a significant percentage of psychiatric institution admissions. The rates of rehospitalization range from 22%-80% [3-5], and these rates are higher during the first months following patient discharge [6]. Rehospitalization is a source of frustration and suffering for both patients and their families. This phenomenon also negatively affects health care systems due to the increasing cost of care [7].

Rehospitalization is influenced by multiple factors and should be assessed within the context of each health care system. Rehospitalization has been utilized as an indicator of whether social networks will accept mentally ill patients and, more recently, as an indicator of the quality of health services. However, there is no consensus on the reliability of this parameter for measuring the quality of care [1,6,8].

The following factors have been associated with the highest risk of rehospitalization: the first episode of mental disorder occurring at a young age, unemployed status, and belonging to a low socio-economic strata [4,5,9-11]. The association between rehospitalization and gender is inconclusive. Several studies have reported a greater risk among men, whereas other studies suggest that the risk is greater among women [9,12]. Additionally, a higher number of previous hospitalizations and a longer duration of hospitalization during the original episode have been associated with a higher risk of rehospitalization [5,13]. Diseases that cause patients to present significant psychotic symptoms, such as schizophrenia and affective disorders, and a history of aggression or symptoms at discharge have been associated with greater rates of rehospitalization [6,12,14,15].

One of the most consistent findings is the association between alcohol dependence, alcohol abuse, or the use of other addictive substances and rehospitalization [16,17]. However, several authors have posited that this association is most likely related to the presence of adverse social and economic conditions. The association between medication adherence and the risk of hospitalization is unclear. The majority of studies have found a relationship; however, some studies have not found an association [5,18,19].

The impact of these factors on rehospitalization varies depending on the time of readmission. Early readmission is defined as rehospitalization that occurs 1-3 months following the first hospitalization, and long-term readmission is defined as rehospitalization that occurs 1-5 years following the first hospitalization [20].

Determining the factors that contribute to the rehospitalization phenomenon may assist in the development of programs and policies to prevent rehospitalization, improve patient care and quality of life, as well as reduce the costs of care, especially in care models with reduced availability of beds and greater restrictions for caring for mentally ill patients [7,8,13].

Therefore, the purpose of this study was to establish the rates of rehospitalization and the factors that were associated with rehospitalization in a psychiatric clinic.

## Methods

A prospective cohort study was conducted between August and December of 2009, with patients who were hospitalized at the Our Lady of Peace Clinic, a private mental health clinic. The clinic attends to adult patients, who were insured mainly through the EPS, *Entidad Promotora de Salud*, (a Health Insurance organization that is part of the Health System in Colombia).

All of the patients had this insurance system that paid for their treatment. After discharge, the patients had follow-ups with EPS associated psychiatrists. When rehospitalization was necessary, the social security system ensured that the patient be admitted or referred to the same clinic.

These hospitalizations were considered the initial hospitalizations. The inclusion criteria included the willingness of patients to provide information over the telephone during a year of follow-up. The exclusion criteria included the unavailability of patients for follow-up after discharge either in person (under hospital control) or by telephone. During this period of recruitment, 419 patients were identified. Of these patients, 24 did not agree to participate in the follow-up and 34 were unable to be contacted at any time after discharge. Therefore, a total of 361 patients were enrolled in the study and followed during a 1-year period to determine whether they were rehospitalized during the follow-up period. This sample size allowed us to reach 80% power due to the model risk ratio. The probability of an event was 0.45. For the independent variables, the R2 was 0.7 and the standard deviation was 4. The hazard ratio (HR) was 2, and the level of significance was 5%. The following variables were measured for patients at baseline: age, sex, education, occupation, marital status, socio-economic strata (a ranking used by the government that classifies houses and properties for the purposes of property taxation. Economic rankings range from one to six. A higher ranking might correlate to a higher income, but is not necessarily the case), the duration of the initial hospitalization, the number of previous hospitalizations, the duration of mental illness (measured as time from first diagnosis), the Axis 1 psychiatric diagnosis, co-morbid medical conditions, the reason for discharge during the initial hospitalization, the number of symptoms, psycho-social support, and adherence to psychiatric treatment. These variables were assessed using the information in the medical history records at the institution. The outcome of interest for the cohort study was a rehospitalization event during the study period. To detect rehospitalization events, the medical history records were reviewed monthly; however, telephone interviews were conducted when this information was not recorded in the medical history. At enrollment, the date of discharge during the initial hospitalization was considered.

For the descriptive component of the study, median, mean, standard deviation, and interquartile range were calculated depending on the symmetry of the distribution (in the case of the continuous variables). The categorical variables were summarized as frequencies and percentages. The frequencies of the categorical variables, depending on the rehospitalization event, were compared using Fisher’s exact test. The medians and means for the continuous variables were compared between the groups of rehospitalized and non-rehospitalized patients using *t*-tests or the sum of the values in a range. The Kaplan-Meier method was utilized to describe the function of time that led up to rehospitalization. Additionally, the frequency of the events was described using incidence rates that were expressed with a corresponding confidence interval of 95%. Depending on the different strata of the categorical variables, the comparison of the survival functions was conducted using the Peto-Peto-Prentice test, taking into account that this test is not susceptible to differential patterns of censure between groups. A multivariate analysis, in which rehospitalization was the outcome of interest, was performed using the Cox proportional hazards model. The model assumptions were evaluated using Schoenfeld residuals plots. For hypothesis testing, a significance level of 5% was used. The statistical analysis was conducted using Stata 11®.

The researchers approached the eligible patients, and invited them to participate in the study. The patients, who accepted it, gave their informed consent. The Ethics Committee of Our Lady of Peace Clinic approved this study.

## Results

Of the 361 patients in the cohort, 217 (60%; 95% CI: 55%-65%) experienced at least one rehospitalization event during the follow-up period (for this study only the first episode was taken into account for the analysis). The distribution of the socio-demographic variables is shown in Table 1. A higher frequency of patients who reported being separated, divorced, or single was found in the group that experienced rehospitalization. The patients who were rehospitalized more often belonged to socio-economic strata 2 and 3 (lower strata, assumed to correspond to lower income), than patients who were not rehospitalized. For the remaining socio-demographic variables, no significant differences were found between groups.

**Table 1:** The distribution of the demographic variables according to rehospitalization events

		**Total sample**	**Rehospitalization**	
			**No**	**Yes**
Age: Mean (standard deviation)		39.4 years (12.4)	39.6 years (12.4)	39.18 (12.5)
Female: Frequency (%)		201 (55.7%)	75 (52.1%)	126 (58%)
Education: Frequency (%)	Illiterate	8 (2.3%)	4 (3%)	4 (1.95)
	Other	5 (1.5%)	2 (1.5%)	3 (1.4%)
	Primary	73 (21%)	32 (24.2%)	41 (19.1%)
	Secondary	136 (39.2%)	48 (36.4%)	88 (40.9%)
	Technical degree	50 (14.4%)	22 (16.7%)	28 (13.0%)
	University degree	75 (21.6%)	24 (18.2%)	51 (23.7%)
Occupation in the last year: Frequency (%)	Unemployed	162 (45.55)	66 (47.1%)	96 (44.4%)
	Employed	149 (41.9%)	62 (44.3%)	87 (40.3%)
	Student	24 (6.7%)	7 (5.0%)	17 (7.9%)
	Retired	21 (5.9%)	5 (3.6%)	16 (7.4%)
Marital status: Frequency (%)*	Married	80 (22.3%)	39 (27.3%)	41 (19.0%)
	Separated/divorced	66 (18.4%)	19 (13.3%)	47 (21.8%)
	Single	170 (47.4%)	64 (44.8%)	106 (49.1%)
	Common-law marriage	43 (12.0%)	21 (14.7%)	22 (10.2%)
Persons they currently live with: Frequency (%)	Institutionalized	9 (2.5%)	3 (2.1%)	6 (2.8%)
	Family members	330 (92.2%)	133 (93.7%)	197 (91.2%)
	Alone	19 (5.3%)	6 (4.2%)	13 (6.0%)
Socio-economic strata: Frequency (%)*	1-2	92 (38.7%)	47 (47.5%)	45 (32.4%)
	3-4	143 (60.1%)	51 (51.5%)	92 (66.2%)
	5-6	3 (1.3%)	1 (1.0%)	2 (1.4%)

*Fisher's exact test, p < 0.0.

**Figure 1:**
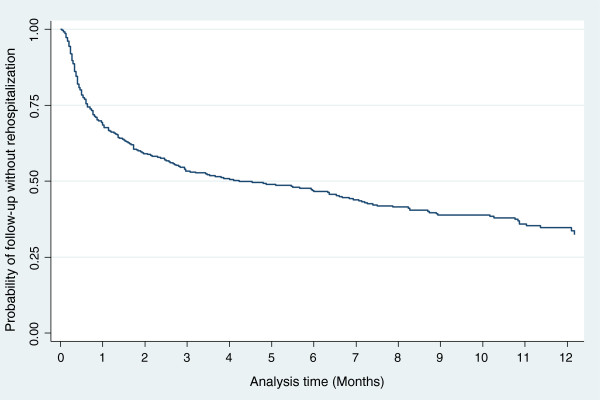
Kaplan-Meier survival function for rehospitalization.

**Figure 2:**
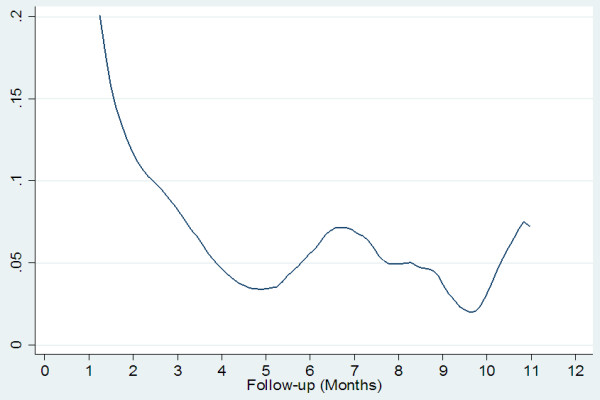
The risk of rehospitalization function (softened with triangular kernel).

Regarding the clinical variables, a greater duration of initial hospitalization and a greater duration of mental illness were found in the patients who were rehospitalized. Additionally, the group of rehospitalized patients had a lower frequency of diagnosed secondary illness and a lower incidence of medical co-morbidity (additionally diagnosed medical conditions that were not the primary reason for consultation). For the rest of the clinical variables, no significant differences were found (Table 2).The rate of rehospitalization was estimated to be 12.4 per 100 patients/months (95% CI: 10.8-14.1). The median survival for rehospitalization was 4.2 months. The Kaplan-Meier survival function is presented in Figure 1.The greatest risk for rehospitalization was observed during the first month after discharge and began to gradually decrease until new increments of risk occurred; however, the risk was not as high as during the initial hospitalization at months 6-7 and 11 (Figure 2).

**Table 2:** The distribution of the clinical variables according to rehospitalization events

		**Total sample**	**Rehospitalization**	
			**No**	**Yes**
Duration of initial hospitalization (median, p25-p75)* (Time from first diagnosis)		10 days (5-14)	8 days (4-12)	10 days (5-15)
#of previous hospitalizations (median, p25-p75)		1 (0-3)	1 (0-2)	1 (0-3)
Duration of illness (median, p25-p75)*		5 years (1-15)	4 years (1-12)	5 years (2-15)
Axis 1 diagnosis: Frequency (%)§	ABD	104 (28.8%)	34 (23.6%)	70 (32.3%)
	Schizophrenia, schizoaffective	86 (23.8%)	34 (23.6%)	52 (24.0%)
	Severe depression	81 (22.4%)	30 (20.8%)	51 (23.5%)
	Substance abuse-related	21 (5.8%)	4 (2.8%)	17 (7.8%)
	Secondary to medical condition	29 (8.0%)	20 (13.9%)	9 (4.2%)
	Other	12 (3.3%)	7 (4.9%)	5 (2.3%)
	Anxiety	28 (7.8%)	15 (10.4%)	13 (6.0%)
Axis 2 diagnosis: Frequency (%)	Group A	13 (3.6%)	4 (2.8%)	9 (4.2%)
	Group B	60 (16.6%)	22 (15.3%)	38 (17.5%)
	Group C	25 (6.9%)	11 (7.6%)	14 (6.5%)
	Intellectual or physical Disability	24 (6.7%)	11 (7.6%)	13 (6.0%)
	Undiagnosed	239 (66.2%)	96 (66.7%)	143 (65.9%)
Psychotropic susbstances consumption: Frequency (%)	Yes	68 (18.8%)	24 (16.7%)	44 (20.3%)
Medical comorbidity: Frequency (%)§	Yes	84 (23.3%)	41 (28.5%)	43 (19.8%)
Cause of hospital discharge: Frequency (%)	Improvement	314 (87.2%)	124 (86.1%)	190 (88.0%)
	Voluntary	41 (11.4%)	19 (13.2%)	22 (10.2%)
	Referral	5 (1.4%)	1 (0.7%)	4 (1.9%)
Number of symptoms at discharge: Frequency (%)	3 symptoms	222 (88.5%)	85 (91.4%)	137 (86.7%)
	4 symptoms	29 (11.6%)	8 (8.6%)	21 (13.3%)
Received ambulatory treatment: Frequency (%)	Never	31 (14.2%)	15 (165%)	16 (12.5%)
	Partially	126 (57.5%)	51 (56.0%)	75 (58.6%)
	Completely	62 (28.3%)	25 (27.5%)	37 (28.9%)

§Fisher's exact test, p < 0.05.

*Test for the sum of the values in a range, p < 0.05.

After applying the Peto-Peto-Prentice test, a significant difference was found between the survival functions for the variables of education, occupation, socio-economic strata, Axis 1 diagnosis, and co-morbidity. The different rehospitalization rates for these variables are presented in Table 3. An increased frequency of rehospitalization was observed in patients with higher educational levels. Regarding occupation, the patients who were students had the highest rehospitalization rates, and the patients who were unemployed had the lowest ones. Regarding marital status, the patients who reported being separated, divorced, or single presented the highest frequencies of rehospitalization. Regarding diagnosis, the highest rates of rehospitalization corresponded to patients with a diagnosis of substance abuse at admission.

**Table 3:** The rehospitalization rates for the variables with significantly different survival functions

**Variable**		**Events**	**Rate**	**95% CI**
Education	Illiterate	4	6.33	2.37 16.85
	Other	3	7.22	2.33 22.40
	Primary	41	9.27	6.83 12.59
	Secondary	88	14.87	12.07 18.33
	Technical degree	28	10.54	7.28 15.27
	University degree	51	18.87	14.34 24.83
Occupation	Unemployed during last year	96	10.71	8.77 13.08
	Employed during last year	87	12.81	10.38 15.81
	Student	17	24.45	15.20 39.33
	Retired	16	17.60	10.78 28.73
Marital Status	Married	41	9.91	7.29 13.45
	Separated/divorced	47	17.24	12.96 22.95
	Single	106	12.94	10.70 15.66
	Common-law marriage	22	9.39	6.18 14.26
Axis 1 Diagnosis	ABD	70	14.07	11.13 17.78
	Schizophrenia, schizoaffective and other	52	11.17	8.51 14.66
	Depression	51	16.69	12.68 21.96
	Related to substance abuse	17	20.51	12.75 33.00
	Secondary to medical condition	9	4.43	2.30 8.51
	Other	5	8.12	3.38 19.51
	Anxiety	13	9.25	5.37 15.94

To determine the variable that was highly predictive of rehospitalization, a stepwise Cox regression was conducted, which included a complete model (this model incorporated the variables with significance levels lower than 0.2 in the univariate analysis) and utilized a withdrawal probability of 0.15. According to the Cox regression, the variables that best predicted the outcome included marital status (patients who reported being separated, divorced, or single were more at risk than patients who reported being married), diagnosis (substance abuse disorders, schizophrenia, bipolar disorder, and depression were associated with a higher risk of rehospitalization than mental disorders that were secondary to medical conditions), discharge circumstances (being referred to another institution for treatment was associated with a higher risk than being discharged due to symptom improvement), education (patients who had completed secondary school were more at risk than patients with a university education), and occupation (students and retired individuals were more at risk than unemployed patients) (Table 4).

**Table 4:** The variables that best predict rehospitalization

**Variable**	**HR**	***P *** **value**	**95% CI**
Single vs. married	1.514	0.057	0.988 2.319
Separated/divorced vs. married	1.989	0.010	1.178 3.358
Substance abuse disorder vs. secondary to medical condition	6.401	0.000	2.662 15.396
Substance abuse disorder vs. secondary to medical condition	2.374	0.003	1.351 4.171
Schizophrenia vs. secondary to medical condition	1.755	0.064	0.969 3.179
Depression vs. secondary to medical condition	2.445	0.003	1.353 4.418
Discharged due to remission vs. discharged due to medical order	9.392	0.000	2.728 2.335
High School vs. university degree	1.529	0.021	1.066 2.192
Student occupation vs. unemployed	2.615	0.003	1.378 4.962
Retired vs. unemployed	2.044	0.019	1.124 3.716

## Discussion

The percentage of patient rehospitalizations in this study was within the higher range of rates that have been reported in other studies, which included rates of up to 80%. This finding is significant due to the impact that rehospitalization has on patients and their families from an emotional perspective and the economic impact of this phenomenon on health care services.

As other studies have reported, the greatest risk of rehospitalization was found during the first month following the initial hospitalization [6].

The variable that demonstrated the greatest association with rehospitalization was the pathological use of psychoactive substances, which is similar to the findings in the majority of studies [17] and further emphasizes the importance of substance abuse interventions to reduce the risk of rehospitalization [21,22]. Similarly, the finding that bipolar disorder, depression, and schizophrenia were associated with a greater probability of rehospitalization in the study time frame had been previously reported [11,14,15].

One of the possible explanations of the greater frequency of separated, divorced, and single patients in the rehospitalization group is the lack of social networks that can support the patient during moments of crisis, which would help prevent hospitalization. However, there could be other factors, as well. This finding has not been reported in other studies.

The finding that rehospitalized patients belonged to higher socio-economic strata contrasts the findings that have been reported in the majority of studies [9-11]. It is possible that lower social strata have less social support and financial resources which in turn might have resulted in them not being able to access medical aid at the same rate, as those with higher incomes. Poor treatment adherence was not associated with a risk of hospitalization, which is in contrast to findings in other studies [19]. This discrepancy may be due to either the short follow-up period or the high frequency of poor adherence to pharmacological treatment. Less than a third of the patients had complete treatment adherence after discharge. This study did not evaluate the type of medication that was administered after discharge. In agreement with other studies, this variable may affect the frequency of rehospitalization. It has been suggested that clozapine may provide a protective effect against rehospitalization among patients with schizophrenia [23,24].

The association between a longer duration of initial hospitalization and rehospitalization may be due to the difficulty in managing patients with chronic and severe psychiatric disorders, or with comorbidity, and less positive responses to treatment, which would explain the longer hospital stays and rehospitalization.

## Conclusions

The rehospitalization rate in our study was as high as reported in other studies. The associated factors with it in this group, may contribute to the design of programs that will reduce the frequency of rehospitalization among patients with mental disorders, in countries like Colombia. Additionally, these results may be useful in developing interventions, such as coping skills training [25], psycho-education [26,27], and community care strategies [28], which have been demonstrated to reduce the frequency of rehospitalization.

## Competing interests

The authors declare that they have no competing interests.

## Authors’ contributions

LEJ participated in the design of the study, the review of the literature, the analysis of the data, and the writing of the manuscript. RS participated in the design of the study and methods, the analysis of the data, and the writing of the manuscript. MIE participated in the collection and analysis of the data and the writing of the manuscript. All authors have read and approved the final manuscript.

## Pre-publication history

The pre-publication history for this paper can be accessed here:

http://www.biomedcentral.com/1471-244X/14/161/prepub
